# Nonspecific chronic low back pain conditions and therapeutic practices in Burkina Faso

**DOI:** 10.4102/sajp.v78i1.1787

**Published:** 2022-09-28

**Authors:** Pegdwendé A. Kaboré, Orokiatou B. Zanga, Bénédicte Schepens

**Affiliations:** 1 Laboratory of Physiology and Biomechanics of Locomotion, Institute of Neuroscience (IoNS), Faculty of Motor Sciences, Université Catholique de Louvain, Louvain-la-Neuve, Belgium; 2 Centre National d’Appareillage Orthopédique du Burkina, Ouagadougou, Burkina Faso; 3 Department of Physical Medicine and Rehabilitation, Centre Hospitalier Universitaire de Bogodogo, Ouagadougou, Burkina Faso

**Keywords:** practitioners, physiotherapy, low-income countries, kinaesphobia, Roland Morris Disability Questionnaire, Fear-Avoidance Beliefs Questionnaire

## Abstract

**Background:**

The management of nonspecific chronic low back pain (NCLBP) is complex because of its multifactorial origin.

**Objectives:**

To investigate NCLBP care by evaluating patients’ condition and therapeutic management of health practitioners.

**Method:**

A cross-sectional survey was carried out among 92 patients with NCLBP, 30 medical practitioners (MP) and 20 physiotherapists (PT) from four public health institutions in Burkina Faso. Patients completed the Visual Analogue Scale, Roland Morris Disability Questionnaire and Fear-Avoidance Beliefs Questionnaire. Practitioners were asked about therapy and continuing professional training.

**Results:**

Pain was moderate to intense for 80% of participants with NCLBP. They were functionally affected and showed fear-avoidance beliefs related to physical and work activities. The majority (97%) of medical practitioners prescribed analgesics and 53% prescribed nonsteroidal anti-inflammatory drugs (NSAIDs). Physiotherapy was the most frequently recommended nonpharmacological treatment. Forty-three per cent of medical practitioners referred to physiotherapy; 20% never did. Physiotherapists practised both passive treatments, such as massage (50%), electrotherapy (55%) and thermotherapy (50%), as well as active treatments, such as general exercises (55%), specific exercises (70%), functional revalidation (50%) and back school (40%). Having had recent continuing professional training and assessing risk factors for chronicity were associated with MPs’ and PTs’ therapeutic choices.

**Conclusion:**

Participants with NCLBP showed fear-avoidance beliefs, correlated with their algo-functional status. Prescribing habits of MPs were drug-based. Treatments by PTs were passive and active. Continuing professional training of healthcare practitioners and assessment of risk factors had a positive impact on therapeutic choices.

**Clinical implications:**

Our study is an invitation to the health care system to improve the relationship between a patient’s NCLBP and therapeutic choices.

## Introduction

Nonspecific chronic low back pain (NCLBP) is characterised by painful symptoms that have persisted for at least 12 weeks, located in the lower part of the spine from the dorso-lumbar to the lumbo-sacral hinge with possible irradiation to the buttocks, the iliac crest, the thigh and occasionally to below the knee (Anaes 2000, Cherin & De Jaeger 2011). Nonspecific chronic low back pain is characterised by the absence of underlying pathology (traumatic, tumour, infectious or inflammatory) and by the fact that even if anatomical lesions can be identified, they cannot explain by themselves the pain and the persistent disability (Berquin & Grisart 2016). The influence of psychosocial factors in the chronicisation process of low back pain (LBP) has been reported (Andersen, Haahr & Frost 2007, Louw, Morris & Grimmer-Somers 2007, Waddell et al. 1993). Indeed, maladaptive beliefs about back pain can result in negative consequences on functioning and on patient prognosis. As these beliefs can originate from family and friends, the media, previous experience and/or health care professionals’ messages, informing and educating the patient (by means of reassurance, explanations of the nonsystematic association of pain and injury, encouragement to get and stay physically active) should be included in therapeutic practice (Demoulin et al. 2016). Models of care including biopsychosocial aspects have been developed to enable therapists to systematically analyse the impact of biological, psychological and social components on the patient and contribute to a more efficient way of managing patients (Danneels et al. 2011).

Current challenges in managing LBP are multiple (economic, socioprofessional, psychological and health related) despite advances in health care (Froud et al. 2014, Waddell et al. 1993). In Africa, the annual prevalence rate of LBP is 57% (Morris et al. 2018), and according to the Global Burden of Disease (GBD) Study, it is the third cause of disability, after major depressive disorders and anaemia (GBD 2015). In Burkina Faso, LBP is the leading cause of consultation in rheumatology, with 20% of consultations of the National Reference Hospital in Ouagadougou (Ouédraogo et al. 2014). Despite the emergence of standard treatment guidelines in some African countries (Ghana in 2010, Namibia in 2011 and Ethiopia in 2014), current therapeutic approaches are still far from international recommendations, as they do not suitably take into account the psychosocial risk factors of LBP (Ahenkorah et al. 2019). To enhance the endorsement of a biopsychosocial approach in Africa, studies on patients’ beliefs and health care practices are required (Ahenkorah et al. 2019). Establishing the profiles of patients with NCLBP and of therapeutic practices is essential in sub-Saharan African countries, as the management of NCLBP is a huge challenge due to low income and other multiple priorities (World Health Organization [WHO] 2012).

Our study sought to investigate patients with NCLBP together with therapeutic practices in Burkina Faso. To do so, profiles of patients with NCLBP were established and the therapeutic practices of medical practitioners and physiotherapists were collected.

## Method

Our cross-sectional survey was carried out in Burkina Faso between February and March 2016 in four public health institutions, in accordance with the Helsinki Declaration (World Medical Association 2013) and STROBE guidelines (Von Elm et al. 2007). The protocol was approved by the Université Catholique de Louvain Ethics Committee (reference number B403201523492).

The files of 150 patients with NCLBP and undergoing medical and/or physiotherapy treatment were selected from four public health institutions in urban areas, including three university teaching hospitals and one national rehabilitation centre (Centre Hospitalier Universitaire Yalgado Ouédraogo, Centre Hospitalier Universitaire Sanou Sourou, Centre Hospitalier Universitaire de Tengandogo, Centre National d’Appareillage Orthopédique du Burkina). Participants had NCLBP without any underlying aetiology for at least 3 months prior to our study. They were over 18 years old and were employed. The final sample size was 92 participants out of a possible 150, as 25 individuals originally identified did not arrive during our survey period, 15 did not wish to take part in our study and 18 were unemployed.

Our survey was carried out face to face. The purpose of our study was explained to the participants, and they gave their informed consent before being enrolled. General sociodemographic and anthropometric information, such as gender, age, height, weight, marital status, number of children, education and lifestyle were first collected, and then three questionnaires were presented to the participants.

The first evaluation was of their pain, which was assessed using the Visual Analogue Scale (VAS) (0 for no pain to 100 for maximum imaginable pain) and classified into four categories: less than 30 for low, 30–50 for moderate, 51–70 for intense and greater than 70 for very intense pain (Wrobel 2007).

The second evaluation was of the functional impact and natural evolution of participants’ back pain, which were assessed using the French version of the Roland Morris Disability Questionnaire-RMDQ (Coste et al. 1993, Roland & Morris 1983). This instrument evaluated the functional impact of NCLBP. The questionnaire was composed of 24 items that examined the effect of pain on activities of daily life (e.g. item 1: ‘I stay at home most of the time because of my back’; item 2: ‘I change position frequently to try and get my back comfortable’; item 3: ‘I walk more slowly than usual because of my back’). Each answer corresponded to 1 point, and a total score was obtained by summing all points. This score varied between 0 (no disability) and 24 (severe disability); the higher the score, the greater the functional disability related to back pain. The French version of the RMDQ has been validated with acceptable psychometric qualities (ICC = 0.89, *p* < 0.00001 and a Kappa coefficient > 0.6) (Coste et al. 1993, Roland & Morris 1983). Clinically speaking, the functional impact of LBP is not present if the score obtained is below 5; the disability is considered as severe if the score is higher than 20, and the minimal important clinically difference is 4 points (Jordan et al. 2006, Kamper et al. 2010, Stratford et al. 1996, Stratford & Riddle 2016).

The third evaluation concerned the fear-avoidance beliefs of movement, which were assessed using the French version of the Fear-Avoidance Beliefs Questionnaire (FABQ) (Chaory et al. 2004, Waddell et al. 1993). The first part of the questionnaire assessed the fears and beliefs in relation to physical activity (FABQphys, e.g. item 2: ‘Physical activity makes my pain worse’; item 4: ‘I should not do physical activities which (might) make my pain worse’), and the second part was in relation to professional activity (FABQwork, e.g. item 6: ‘My pain was caused by my work or by an accident at work’; item: 7: ‘My work aggravated my pain’). The questionnaire was made up of 16 items (4 items for FABQphys, 7 for FABQwork and 5 illusory items) rated from 0 (‘I completely disagree’) to 6 (‘I completely agree’). Patients circled the number that best illustrated their degree of agreement about what they experienced and what affected or could affect their back during activities. The FABQphys score was obtained by adding items 2, 3, 4 and 5 (maximum score of 24), and the FABQwork score was obtained by adding items 6, 7, 9, 10, 11, 12 and 15 (maximum score of 42). The psychometric properties of the French version of the FABQ were acceptable, as the test–retest reliability was good, with ICC values of 0.88 and 0.72 for FABQphys and FABQwork, respectively (Chaory et al. 2004). In addition to measuring fear-avoidance beliefs, the FABQ questionnaire allows the prediction of the evolution of the patient’s clinical symptoms (Wertli et al. 2014). When the FABQphys score is higher than 14, the beliefs are considered to have strong clinical implications, such as increased disability (George, Fritz & Childs 2008, Waddell et al. 1993) For the FABQwork, the symptoms improved more rapidly for patients with a score lower than 20 compared to those with a score higher than 20. Patients with a score higher than 29 show persistence of symptoms after 6 months and frequent absences from work despite rehabilitation (Fritz & George 2002, George et al. 2008).

### Health practitioners’ survey

Our survey exploring the care given by MPs and PTs was carried out in physical medicine, neurology, rheumatology, general medicine and medical emergency services, working in the four institutions mentioned above. The inclusion criteria requested everyday practice of patients with NCLBP. Two custom-made questionnaires, one for MP and one for PT, were developed according to each profession’s activity. The care given to patients was explored based on evidence from the literature, taking into account the pharmacological, physical and psychological dimensions of NCLBP treatment (Airaksinen et al. 2006, Kamper et al. 2014). Beforehand, both questionnaires were administered to two MPs and two PTs to evaluate the completion time (5 min) and make the necessary corrections ascertaining the ease of completion of the questionnaires. A note presenting the main goal of our study was given to the practitioners before they completed the questionnaires themselves. A voluntary nonprobability sampling technique was used.

### Medical practitioners’ survey

Thirty medical practitioners agreed to participate. The survey included questions about professional characteristics (gender, age, duration of practice, number of patients with NCLBP consulted per month, any specific training) and closed questions about the frequency (never, sometimes or frequently) of their prescribing habits (pharmacological prescriptions: analgesics, nonsteroidal anti-inflammatory drugs, myorelaxant drugs, antidepressant drugs, steroid infiltrations; non-pharmacological prescriptions: physiotherapy, back school, psychotherapy, cognitive behavioural therapy, rest, lumbar belt, acupuncture, osteotherapy). Medical practitioners were also asked if they evaluated the risk factors of lumbar pain chronicity when they first met their patients and precisely how they did this.

### Physiotherapists’ survey

All physiotherapists (*n* = 20) working in the four institutions agreed to participate in our study. The questionnaire included professional characteristics (gender, age, duration of practice, number of patients with NCLBP treated per month, recent continuing professional training) and closed questions about the frequency (never, sometimes or frequently) of their treatment techniques (massage, electrotherapy, thermotherapy, general exercises, specific exercises, functional revalidation, back school, psychotherapy). The physiotherapists were also asked if they evaluated the factors of risk of lumbar pain chronicity when they first met their patients and precisely how they did it.

### Statistical analysis

Descriptive statistics and frequency tables were produced for all variables (SPSS 26.0). The total scores for VAS, RMDQ, FABQphys and FABQwork displayed a normal distribution. The distribution of variables was verified by the Kolmogorov–Smirnov normality test (*p* > 0.05). Pearson and Spearman correlations were used to analyse the relationships between VAS, RMDQ and FABQ scores and between these variables and sociodemographic characteristics. The chi-square and Fisher’s tests were used to analyse the dependence between the professional characteristics and prescribing and/or treatment habits. The level of significance was set at 0.05.

### Ethical considerations

The study protocol was approved by the Université Catholique de Louvain Ethics Committee (ref. no. B403201523492). The purpose of our study was explained to the participants and their informed consent was obtained before filling out the questionnaires.

## Results

### Patients

The majority of the NCLBP participants were female (60%, Table 1). The age was 47 ± 12 years (mean ± SD), and only 16% were over 60 years old. The BMI was 27.4 ± 5.4 kg/m^2^; 35% of the participants were overweight and 29% obese. Only 3% practised regular physical activity, 33% were alcohol consumers and 23% were smokers. The majority (74%) was either married or living with a partner, 86% had at least one child and 82% had at least a primary school education.

**TABLE 1:** Characteristics of patients (*n* = 92).

Variables	%	Mean ± SD	Min – Max
Age (years)	-	47±12	25–75
BMI (kg/m^2^)	-	27.4±5.4	16.7–41.3
**Gender**			
Male	40	-	-
**Age range (years)**			
< 30	5	-	-
30–40	31	-	-
41–50	29	-	-
51–60	19	-	-
> 60	16	-	-
**BMI range (kg/m^2^)**			
< Normal	4	-	-
Normal	32	-	-
Overweight	35	-	-
Obese	29	-	-
**Regular physical activities**			
Practice	3	-	-
**Alcohol**		-	-
Nonconsumer	67	-	-
**Smoking**			
Nonsmoker	77	-	-
**Marital status**			
Single	17	-	-
Married or cohabitation	74	-	-
Divorced or widower	9	-	-
**Number of children**			
0	14	-	-
1–2	32	-	-
3–4	31	-	-
≥ 5	23	-	-
**Level of education**			
Unschooled	18	-	-
Primary	16	-	-
Secondary	37	-	-
College or university	29	-	-

SD, standard deviation; Min, minimal value; max, maximal value.

**FIGURE 1:**
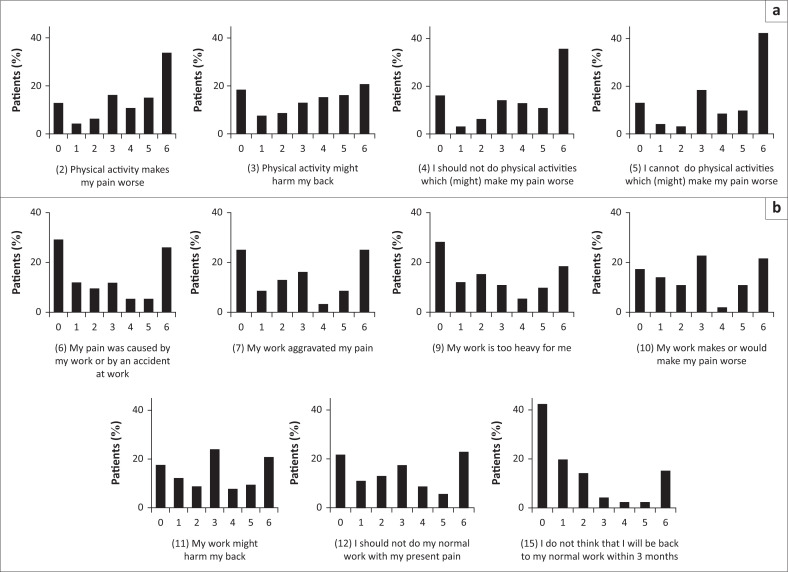
Distribution among the patients of responses by item. (a) FAQBphys, including fears and beliefs related to physical activity (items 2, 3, 4 and 5); (b) FABQwork, including fears and beliefs related to work (items 6, 7, 9, 10, 11, 12 and 15). For each item, response categories range from 0 (‘I completely disagree’) to 6 (‘I completely agree’).

The mean score of the VAS was 56 ± 14 (mean ± SD, Table 2). The pain was considered low to moderate for 34% (6% and 28%, respectively) and intense to very intense for 66% (52% and 14%, respectively). The mean score of the RMDQ was 11 ± 6 (Table 2), and more than 75% of patients considered that the low back pain impacted their functionality. The disability was considered severe for 13% of them. The mean score of FABQphys and FABQwork was respectively 15 ± 6 and 19 ± 12 (Table 2). The FABQphys score was high (> 16) for half of the patients, and the FABQwork was high (> 27.8) for more than 25% of the participants. Figure 1 presents the distribution of the FABQ scores per items. For the FABQphys, the most frequently chosen category was 6 (‘I completely agree’); this was true for the four items (Figure 1a). The responses of the FABQwork were more distributed, as the same proportion of responses were observed: ‘I completely disagree’ (0) and ‘I completely agree’ (6) for items 6, 7, 10, 11 and 12. Notably, the most frequently chosen category was 0 for items 9 and 15.

**TABLE 2:** Visual Analogue Scale, Roland Morris Disability Questionnaire and Fear-Avoidance Belief Questionnaire scores of patients (*n* = 92).

Score	Min – Max	Mean	SD	25th	50th	75th
VAS	30–90	56	14	45.0	55.0	66.5
RMDQ	1–24	11	6	5.3	10.5	13.8
FABQphys	0–24	15	6	12.0	16.0	20.0
FABQwork	0–42	19	12	8.5	18.0	27.8

VAS, Visual Analogue Scale; RMDQ, Roland Morris Disability Questionnaire; FABQphys, Fear-Avoidance Belief Questionnaire related to physical activity; FABQwork, Fear-Avoidance Belief Questionnaire related to work; 25th, percentile 25; 50th, percentile 50; 75th, percentile 75.

Other indications: see Table 1.

**FIGURE 2:**
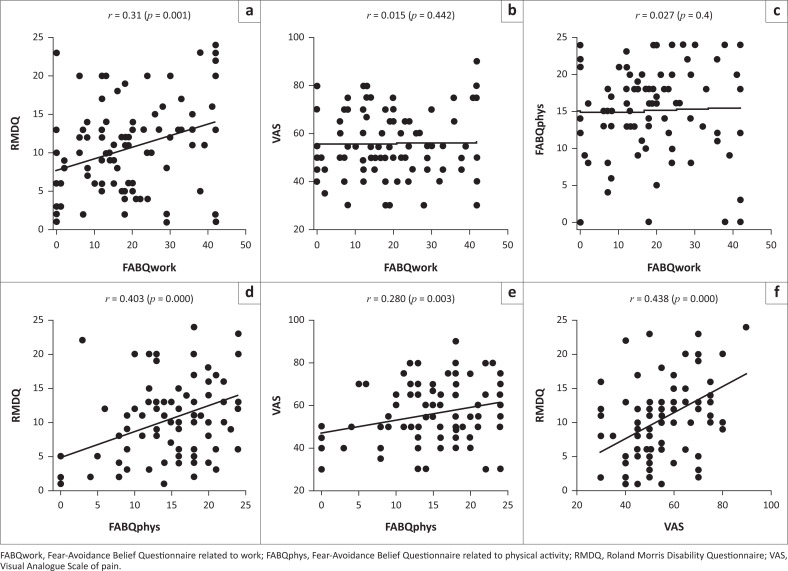
Correlations between patient scores, including correlation coefficients and level of significance.

There was a correlation between FABQwork and RMDQ (*r* = 0.31, *p* = 0.001), FABQphys and RMDQ (*r* = 0.403, *p* = 0.000), FABQphys and VAS (*r* = 0.280, *p* = 0.003) and RMDQ and VAS (*r* = 0.438, *p* = 0.000) scores (Figure 2).

### Practitioners

#### Medical practitioners

Thirty medical practitioners participated, who were mostly male (77%, Table 3). Thirty per cent had more than 10 years of professional experience. A majority (57%) consulted or treated more than 10 patients with NCLBP per month. A minority had continuing professional training on NCLBP (27%) during the previous 3 years. Fifty-seven per cent assessed the risk factors for chronic disease (Table 3). Regarding the method of evaluation, all MPs who answered positively to this question mentioned doing this orally, and it was not based on a specific questionnaire.

**TABLE 3:** Characteristics of health practitioners (medical practitioners and physiotherapists).

Variables	MPs (*n* = 30)	PTs (*n* = 20)
	%	%
**Gender**		
Male	77	70
**Year of work experience**		
> 10 years	30	75
**Number of patients with NCLBP consulted or treated**		
≥ 10 patients per month	57	75
**Continuing professional training received recently**		
Yes	27	40
**Assessment of chronicity factors**		
Yes	57	40

NCLBP, nonspecific chronic low back pain; MPs, medical practitioners; PTs, physiotherapists.

#### Prescribing habits and relationships with professional characteristics of medical practitioners

**FIGURE 3:**
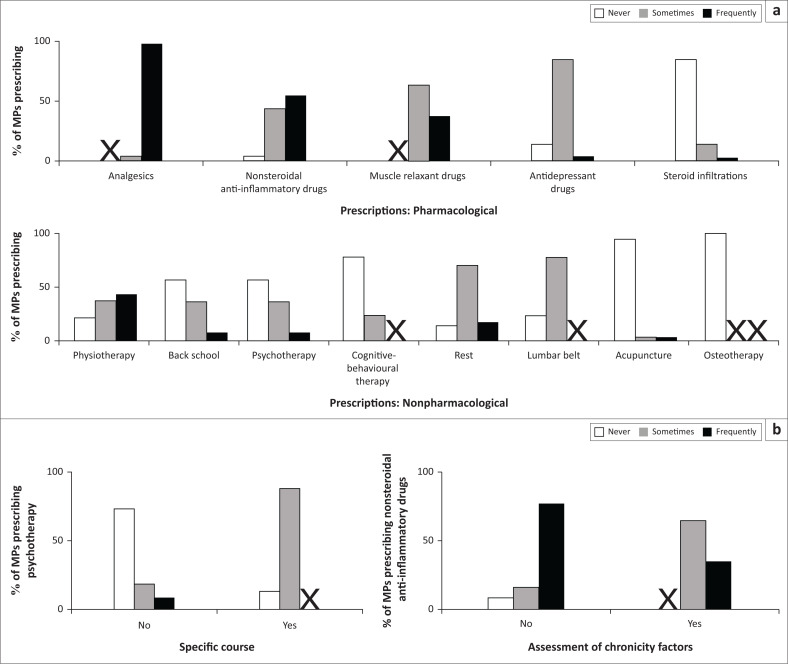
Distribution of medical practitioners (MPs) prescribing habits and relationships with professional characteristics. (a) Prescriptions: pharmacological and nonpharmacological. (b) Relationship between professional characteristics and prescribing habits. Left: specific course and psychotherapy prescription (X^2^ = 12.171, *df* = 2, *p* = 0.002); right: assessment of chronicity factors and NSAID prescriptions (X^2^ = 7.837, *df* = 2, *p* = 0.02). The ‘X’ indicates when no answer was observed in the category.

Analgesics were the most common drugs prescribed (97%), followed by nonsteroidal anti-inflammatory drugs (53%) and muscle relaxants (37%, Figure 3a). Antidepressant medications and corticosteroids were frequently prescribed or injected by 3% of MPs (Figure 3a). Physiotherapy was the most frequently prescribed treatment (43%); however, 20% of MPs said they never wrote referrals for physiotherapy (Figure 3a). Back school (7%) and psychotherapy (7%) were frequently prescribed by a minority of MPs. Rest (17%) and acupuncture (3%) were frequently prescribed.

Having had recent continuing professional training was related to therapeutic choices (Figure 3b). Prescription of psychotherapy was strongly related to having received at least one continuing professional training in the past 3 years (*p* = 0.002): 87% of MPs who did not receive any continuing professional training declared they sometimes wrote referrals for psychotherapy for their patients (Figure 3b). There was a relationship between NSAID’s prescription and assessing NCLBP chronicity risk factors (*p* = 0.02): 77% of MPs who did not assess for chronicity factors frequently prescribed NSAID (Figure 3b).

#### Physiotherapists

Twenty physiotherapists participated, who were mostly male (70%, Table 3). Seventy-five per cent had more than 10 years of professional experience. A majority of PTs (75%) consulted more than 10 patients with NCLBP per month (Table 3). A minority (40%) had continuing professional training on LBP during the past 3 years. A minority (40%) of PTs assessed the risk factors for chronic disease (Table 3). Regarding the method of evaluation, all PTs who answered positively to this question mentioned doing it orally and not based on a specific questionnaire.

#### Practice habits and relationships with professional characteristics of physiotherapists

**FIGURE 4:**
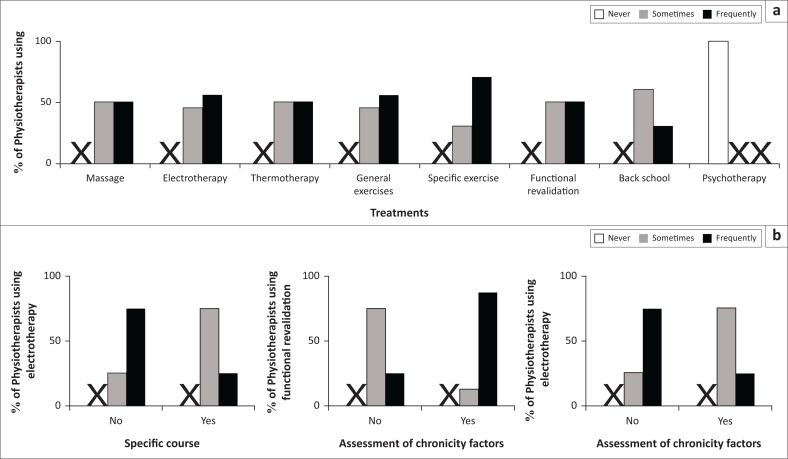
Distribution of physiotherapists’ practice habits and relationships with professional characteristics. (a) Treatment habits. (b) Relationship between professional characteristics and physiotherapy treatment habits. Left: specific course and electrotherapy (*p* = 0.04); middle: assessment of chronicity factors and functional revalidation (*p* = 0.01).Right: assessment of chronicity factors and electrotherapy (*p* = 0.04). The ‘X’ indicates when no answer was observed in the category.

Massage, electro- and thermotherapies were frequently performed by about one half of PTs (50%, 55% and 50%, respectively, Figure 4a). General and specific exercises were frequently implemented by the majority of PTs (55% and 70%, respectively, Figure 4a). Functional revalidation and back school were frequently implemented by about one-half of PTs (50% and 40%, respectively, Figure 4a). No physiotherapists declared practising psychotherapy (Figure 4a).

The practice of electrotherapy was related to having received recent continuing professional training (*p* = 0.04); 75% of PTs who did not receive any specific training frequently treated their patients using electrotherapy (Figure 4b). The practice of electrotherapy was linked to the assessment of risk factors for the chronicity of NCLBP (*p* = 0.04); 75% of PTs who did not assess the risk factors for chronicity frequently practised electrotherapy (Figure 4b). There was a relationship between the practice of functional revalidation and evaluating the risk factors of chronicity (*p* = 0.01); 88% of PTs who assessed chronicity factors frequently included functional revalidation as a treatment (Figure 4b).

## Discussion

The aims of our study were to investigate NCLBP care by evaluating the patients’ condition and the therapeutic management of health practitioners.

One-quarter of NCLBP patients had a FABQwork score close to the threshold of 29, and more than 50% had a FABQphys higher than 16. According to evidence in the literature, these high scores predict persistence of symptoms and disability in individuals with NCLBP after 6 months, despite physiotherapy treatment (George et al. 2008). Similar results have been reported showing that a high FABQwork score was a predictor of work absences and job losses in patients with NCLBP, while the FABQphys score predicts functional disabilities (Waddell et al. 1993). The use of FABQ scores could be used to target therapies, that is, patients with a high FABQwork score (> 29) will benefit from specific attention to multidisciplinary management integrating specific return-to-work programmes, while attention should be redirected to functional aspects of daily living for patients with a high FABQphys score (> 14). These clinical predictions have the advantages of distinguishing subgroups and allow the appreciation of the extent and severity of pain of patients with NCLBP, their functional disorders and the implications of fear-avoidance false beliefs, but their levels of evidence are still low (George et al. 2008).

The scores of the FABQphys indicated quite homogeneous deleterious beliefs related to physical activity (score of 6: ‘I completely disagree’ for a majority of patients). These false beliefs scores are associated with a risk of developing functional disorders such as kinaesiophobia, that is, fear of movement, which should be specifically taken into account when implementing treatment guidelines (Demoulin et al. 2016, Waddell et al. 1993).

In contrast, the beliefs related to work activity appear to be more mixed, as the distribution of patients’ beliefs for each item of the FAQBWork is more balanced between strong false beliefs (score of 0: ‘I completely agree’) and no false beliefs (score of 6: ‘I completely disagree’). This disparity among the participants supports the necessity to adapt each treatment to each patient by ensuring that useful information for a better understanding of the pathology and its consequences is provided to each patient (fight against maladaptive beliefs).

As in other studies, it was observed that functional disorders and pain increase with false beliefs (George, Fritz & McNeil 2006, Chung, Hur & Lee 2013, Igwesi-Chidobe et al. 2017, Tarimo & Diener 2017). In South Korea, the intensity of fear-avoidance related to both physical and professional activities has been shown to be related to functional disability (Chung et al. 2013). In the United States of America, the increase of fear and false beliefs was proportional to the change in the intensity of pain and disability (George et al. 2006). In Nigeria, beliefs of fear-avoidance and perceptions of illness were important factors associated with NCLBP disability (Igwesi-Chidobe et al. 2017). In Malawi, 93% of the LBP population showed kinaesiophobia in daily activities (Tarimo & Diener 2017). Taking into account the perceptions and beliefs of patients is particularly important in sub-Saharan countries, where most health care is the responsibility of patients and/or their families, due to the lack of infrastructure and/or trained health professionals (Sogbossi et al 2021). Faced with pain and its repercussions on their quality of life, patients undertake steps to grasp and understand the ‘meaning of this evil’, hoping to find healing according to their income, environment and beliefs. These attitudes can lead to therapeutic choices between ‘modern’ and ‘traditional’ treatments, such as consulting marabous, witch doctors and prayer groups. It is not uncommon in Burkina Faso to hear from patients with NCLBP that they are victims of a spell from colleagues or from someone jealous of their success.

Interestingly, the level of pain observed here was moderate to intense for the majority (80%) but very intense only for a minority (14%). This threshold drop could partly be explained by the fact that in patients with NCLBP, the persistence of pain can result in functional and structural changes of the nervous system, leading to a decrease of the pain threshold (Nijs et al. 2014). Pain and disability are no longer solely influenced by organic pathologies but also by psychosocial factors, hence the interest in screening for false beliefs such as fear-avoidance in patients with NCLBP.

Finally, the majority of patients presented with functional disabilities (RMDQ > 5.3) (Table 2). As a score of 4 is the limit between functional and dysfunctional in LBP patients (Kamper et al. 2010, Stratford et al., 1996, Stratford & Riddle, 2016), functional rehabilitation should be integrated into multidisciplinary management to improve their quality of life.

It was observed that pharmacological treatments are predominantly used by MPs in the management of NCLBP. A study carried out in the public primary health care facilities in South Africa showed similar trends: analgesics were the main treatments for 93% of NCLBP sufferers, and physiotherapy was rare (16%) (Major-Helsloot et al. 2014). Our study’s results on drugs prescription are mainly in accordance with the guidelines’ recommendations: analgesics at level 1 and short-term use of NSAIDs and other analgesics (Kuijpers et al. 2011, Qaseem et al. 2017, Van Wambeke et al. 2017). But it was observed that corticosteroid injections were frequently performed by some MPs, although their long-term effectiveness has not been demonstrated in the management of NCLBP (Kuijpers et al. 2011, Qaseem et al. 2017, Van Wambeke et al. 2017). Even if pharmacological treatment has its place in the multidimensional management of NCLBP, the strong preference for it compared to other forms of treatment (physical, psychological, educational) does not match the evidence based on practice and does not allow for optimal recovery of patients (Airaksinen et al. 2006, Kamper et al. 2014).

Concerning nonpharmacological prescriptions, physiotherapy is prescribed by a majority of MPs, but one fifth of MPs never wrote referrals for physiotherapy; this does not correspond to current recommendations where physiotherapy occupies a prominent place in NCLBP management (Van Middelkoop et al. 2011). Our results also show the scarcity of prescriptions taking into account psychosocial risk factors, such as cognitive-behavioural therapy, psychotherapy and back school, while these kinds of approaches are more and more promoted in the management of false beliefs with a tendency towards kinaesiophobia (Demoulin et al. 2016). Perhaps the lack of referral for these techniques observed in our sample in Burkina Faso is due to the absence of professionals specifically trained for this purpose and/or to the limited access to such training. This should be further analysed.

The so-called passive interventions (i.e. not requiring active participation from the patients, such as massage, electrotherapy and thermotherapy) and interventions requiring more active participation from the patient (such as general and specific exercises, functional revalidation, psychotherapy and back school) are both used by PTs. But only a minority of the PTs (40%) performed back school techniques, although this kind of therapy includes a global approach to patients and of their environments. The use of passive techniques is frequent in Africa (South Africa) (Major-Helsloot et al. 2014), but this is changing, as in Ghana where the techniques have become more active (Oppong-Yeboah & May 2014). Note that although these active techniques are recommended, they remain biomedical approaches and their efficiency seems limited when not associated with other types of therapies that consider psychosocial factors (Demoulin et al. 2016, Van Wambeke et al. 2017).

The therapeutic choices made by practitioners seem related to continuing professional training. Medical practitioners having had the opportunity of specific training are more likely to prescribe psychotherapy, which is an effective treatment when included in multidisciplinary patient management (Van Wambeke et al. 2017). The same is true for PTs, who focus less on electrotherapy if they have recently received specific training. These observations reinforce and enlarge the importance of continuing professional training for health practitioners to improve the quality of their care, to update their knowledge and to avoid routine pitfalls.

Concerning the evaluation of LBP chronicity factors, the MPs who assess them prescribe NSAIDs less often compared to those who do not assess chronicity factors. A similar observation is true for PTs: the PTs who assess LBP chronicity factors practise more functional revalidation and fewer electrotherapy techniques, compared to those who do not assess chronicity factors. The use of a standardised questionnaire seems not to be a common practice and should be promoted. The advantages of using a questionnaire are, on the one hand, to objectify the patients’ beliefs and follow their progress (Ogunlana et al. 2015). On the other hand, the risk factors can be discussed and communicated to colleagues to ensure best care. The absence of such a tool could alter diagnoses and reduce the effectiveness of the interventions. The poor use of such approaches observed here could be explained by the diversity of languages in Burkina Faso (even if French is the official language), but also by the heavy workload of health practitioners. In Burkina Faso, the ratio of medical practitioners to the population is estimated as 1 per 22 000, while the minimum standard set by the WHO is one MP per 10 000 inhabitants (WHO 2010). The deficit is even greater for the PTs – less than 50 PTs and 150 PT assistants for the whole country.

### Strengths and limitations

Our study shows, for the first time in Burkina Faso, the potential inadequacy in the care and management of NCLBP. It also shows the importance of continuing professional training of health care practitioners in the management of patients with NCLBP.

It is acknowledged that our study cannot be generalised to the whole country, as only patients attending urban health care institutions were surveyed. But even if the profile of the NCLBP patients (pain, functional disorders, work and physical activity beliefs) and of the practices of healthcare practitioners presented here needs to be confirmed by larger studies, our preliminary study could potentially help to implement education programmes and guidelines.

### Implications and recommendations

Our study is an invitation to the health care system to improve the management of NCLBP by health practitioners. This could be done by promoting continuing professional training of MPs and PTs, including giving special attention to existing maladaptive beliefs on back pain.

We recommend a systematic assessment of psychosocial risk factors to be included in the management of NCLBP in Burkina Faso. Future training courses should, for example, include the FABQ or a similar questionnaire as a basic tool for screening psychosocial risk factors.

## Conclusion

We have shown that patients with nonspecific chronic low back pain in Burkina Faso hospitals present with functional disorders and various fear-avoidance beliefs which are associated with their functional disorders and pain. The prescribing habits of medical practitioners were mainly drug-based. The treatments of physiotherapists were both passive and active. Our study also shows that, when present, continuing professional training of healthcare practitioners and assessment of the risk factors of chronic disease had a positive impact on therapeutic choices.
